# Status and Recommendations of Technological and Data-Driven Innovations in Cancer Care: Focus Group Study

**DOI:** 10.2196/22034

**Published:** 2020-12-15

**Authors:** Haridimos Kondylakis, Cristian Axenie, Dhundy (Kiran) Bastola, Dimitrios G Katehakis, Angelina Kouroubali, Daria Kurz, Nekane Larburu, Iván Macía, Roma Maguire, Christos Maramis, Kostas Marias, Philip Morrow, Naiara Muro, Francisco José Núñez-Benjumea, Andrik Rampun, Octavio Rivera-Romero, Bryan Scotney, Gabriel Signorelli, Hui Wang, Manolis Tsiknakis, Reyer Zwiggelaar

**Affiliations:** 1 FORTH-ICS Heraklion Greece; 2 Audi Konfuzius-Institut Ingolstadt Lab Technische Hochschule Ingolstadt Ingolstadt Germany; 3 School of Interdisciplinary Informatics University of Nebraska Omaha, NE United States; 4 Interdisziplinäres Brustzentrum Helios Klinikum München West Munich Germany; 5 Vicomtech, Health Research Institute San Sebastian Spain; 6 University of Strathclyde Glasgow United Kingdom; 7 eHealth Lab Institute of Applied Biosciences - Centre for Research & Technology Hellas Thessaloniki Greece; 8 School of Computing Ulster University Newtownabbey United Kingdom; 9 Salumedia Labs & Adhera Health, Inc Sevilla Spain; 10 Academic Unit of Radiology, Department of Infection, Immunity and Cardiovascular Disease University of Sheffield Sheffield United Kingdom; 11 Universidad de Sevilla Seville Spain; 12 School of Computing and Engineering University of West London London United Kingdom; 13 Department of Computer Science Aberystwyth University Aberystwyth United Kingdom

**Keywords:** neoplasms, inventions, data-driven science

## Abstract

**Background:**

The status of the data-driven management of cancer care as well as the challenges, opportunities, and recommendations aimed at accelerating the rate of progress in this field are topics of great interest. Two international workshops, one conducted in June 2019 in Cordoba, Spain, and one in October 2019 in Athens, Greece, were organized by four Horizon 2020 (H2020) European Union (EU)–funded projects: BOUNCE, CATCH ITN, DESIREE, and MyPal. The issues covered included patient engagement, knowledge and data-driven decision support systems, patient journey, rehabilitation, personalized diagnosis, trust, assessment of guidelines, and interoperability of information and communication technology (ICT) platforms. A series of recommendations was provided as the complex landscape of data-driven technical innovation in cancer care was portrayed.

**Objective:**

This study aims to provide information on the current state of the art of technology and data-driven innovations for the management of cancer care through the work of four EU H2020–funded projects.

**Methods:**

Two international workshops on ICT in the management of cancer care were held, and several topics were identified through discussion among the participants. A focus group was formulated after the second workshop, in which the status of technological and data-driven cancer management as well as the challenges, opportunities, and recommendations in this area were collected and analyzed.

**Results:**

Technical and data-driven innovations provide promising tools for the management of cancer care. However, several challenges must be successfully addressed, such as patient engagement, interoperability of ICT-based systems, knowledge management, and trust. This paper analyzes these challenges, which can be opportunities for further research and practical implementation and can provide practical recommendations for future work.

**Conclusions:**

Technology and data-driven innovations are becoming an integral part of cancer care management. In this process, specific challenges need to be addressed, such as increasing trust and engaging the whole stakeholder ecosystem, to fully benefit from these innovations.

## Introduction

### Background

The morbidity and mortality associated with cancer are rapidly increasing globally because of population growth and aging, reflecting the changes in the prevalence and distribution of major risk factors of cancer [1]. These global trends have resulted in more people living with or beyond cancer. As such, there is a greater need to improve and optimize cancer care services throughout diagnosis, treatment, rehabilitation, and end-of-life care. Modern technologies, often enabled by the availability of big data and advanced analytics, have demonstrated the potential to enhance the current level of quality of cancer care, for example by improving information access, informing and sharing clinical decision making with patients, and facilitating communication and support for coliving with the illness.

Pioneering research is now being conducted in the field of cancer care technology, resulting in the development of novel solutions to a diverse spectrum of problems in this area. However, the process of evaluating these innovations and their operation within a *real-world* context is at a less advanced stage. The clinical assessment of technology poses many challenges and is influenced by numerous variables. As a result, there is an emphasis on the early evaluation of technology by including key stakeholders throughout the design and development phases (sometimes referred to as a cocreation process).

### Objectives

Evidently, as researchers involved in the creation of novel technologies for cancer care, we must consider and share both the innovative concepts being developed as well as how they are being assessed and accepted in real-world or clinical settings.

To this end, an international workshop was convened to consider the current status of technological and data-driven innovations in cancer care, to identify key challenges and opportunities, and to formulate recommendations aimed at accelerating the rate of progress in the data-driven management of cancer. The two workshop instances led to a series of publications. This paper discusses key topics arising from the workshops and subsequent discussions among their participants.

## Methods

An international expert consensus-building workshop named Tech4Cancer [2] was held in two instances; the first was held in June 2019 in Cordoba, Spain, along with the computer-based medical systems [3] international conference, and the second was held in October 2019 in Athens, Greece, along with the Institute of Electrical and Electronics Engineers (IEEE) Bioinformatics and Bioengineering [4] international conference. The workshops attracted significant international participation; more than 40 participants attended these events, where they focused on discussing global trends for technological and data-driven innovations in cancer care.

The workshop was supported by four European Union (EU) projects, namely BOUNCE, CATCH ITN, DESIREE, and MyPal, funded by Horizon 2020 (H2020). The BOUNCE project [5,6] considers clinical, cancer-related biological, lifestyle, and psychosocial parameters to predict individual resilience trajectories throughout the cancer continuum. Eventually, the target is to increase resilience in breast cancer survivors and help them remain in the workforce and have a better quality of life. DESIREE [7], on the other hand, aims to provide a web-based software ecosystem for personalized, collaborative, and multidisciplinary management of primary breast cancer by specialized breast units, from diagnosis to therapy and follow-up.

MyPal [8] aims to foster palliative care for people with cancer by leveraging patient-reported outcome (PROM) systems through their adaptation to the personal needs of the person with cancer and his or her caregivers [9]. In this regard, MyPal designed two novel eHealth interventions based on electronic PROMs to offer advanced palliative care services to adult patients with cancer and children with cancer. MyPal interventions are delivered via a sophisticated eHealth platform developed by the project. The interventions and the platform itself will be assessed through two multicenter clinical studies (one for the case of adults and one for the case of children), which will take place throughout Europe. CATCH ITN [10] is a PhD training network, with academic and industry partners across Ireland, Spain, and Denmark. Research within CATCH ITN focuses on the development and use of technology to improve the quality of life of individuals living with and beyond cancer.

The primary deliverable from this workshop was a set of articles that summarize key issues already published by IEEE. During the paper presentation, several topics were identified through discussion among the participants. After the second instance of the workshop, participants were invited to participate in a focus group discussing status, challenges, opportunities, and recommendations in the area of technological and data-driven cancer management; the outcome is reported in this study.

The writing group met by teleconference, and participants were asked to propose topics according to their interest and expertise and to select topics that they could actively contribute to. Several topics depicting challenges, opportunities, and recommendations were selected. Subsequently, leaders for each topic were identified and a structure was proposed. A formal consensus process was not used; however, the structured and open discussions did not reveal any fundamental disagreements about the nature of the topics, although the discussions supported the refinement and specificity of topics. Once all contributions were collected, a homogenization and integration process led to a final draft, where all participants commented and discussed, leading to the final submitted version.

## Results

### Patient Engagement and Participatory Design

A fundamental requirement for the effectiveness of any eHealth intervention, including interventions addressed to patients with cancer and survivors, is a certain level of *engagement* by the patient [11], who is the main beneficiary of the intervention [12]. The lack of engagement of patients with eHealth interventions is linked to low motivation and adherence to the intervention, leading to high dropout rates and eventually affecting treatment outcomes and effectiveness [13]. The issue of patient engagement has cast a shadow over the enthusiasm for the potential of eHealth [14].

Formulating a concrete definition of patient engagement in the context of eHealth is a challenge by itself [14], as there is a lack of consensus on what the term engagement entails and because different fields (eg, computer science, psychology, and behavioral health) conceptualize it differently. A working definition comes from the Canadian Institutes for Health Research in the framework of the Strategy for Patient-Oriented Research: patient engagement is the meaningful and active collaboration of the patient in governance, priority setting, research, and knowledge translation [15]. Some researchers have defined engagement in terms of the level of *activation* of the patient in the management of their own health [16]. Certain definitions rely on psychological processes related to user perceptions and experience, whereas others consider engagement as a purely behavioral construct, synonymous with intervention usage [17]. Consequently, engagement is often used interchangeably with *adherence*, which refers to whether the intervention is used as intended by its developers [18,19]. This is the definition of patient engagement that is primarily considered in the context of this study.

The situation is not significantly better when it comes to the assessment of patient engagement. The lack of consensus in the conceptualization of user engagement makes the design of appropriate *universal* or even *widely applicable* assessment instruments difficult. Instead, most research efforts propose solutions for assessing user engagement for very specific purposes, modalities, and contexts pertaining to eHealth [11,20,21]. Focusing on mobile health (mHealth), a prominent subfield of eHealth, a recent scoping review analyzed 41 studies and developed a library of 7 analytic indicators to evaluate effective engagement with consumer mHealth apps for chronic conditions, namely the (1) number of measures recorded, (2) frequency of interactions logged, (3) number of features accessed, (4) number of log-ins or sessions logged, (5) number of modules or lessons started or completed, (6) time spent engaging with the app, and (7) number or content of pages accessed [22]; this work is of particular relevance to care for cancer, which currently tends to be considered a chronic disease [23].

The scientific literature sheds some light on the techniques that have been employed to achieve, maintain, and improve patient engagement. According to a psychology study [24], patient engagement techniques are categorized as follows:

1. Behavioral techniques, such as motivational interviewing, goal setting, and planning, which are related to patient actions when managing their health condition.
2. Cognitive techniques, such as question-asking tasks and psychoeducation sessions, which are related to patient thoughts and received information concerning their health condition.
3. Emotional techniques, such as positive psychology exercises and expressive writing tasks, which are related to experienced patient feelings and emotions when adjusting to their new health condition.

Most interventions developed for older adults employ patient engagement techniques from behavioral and/or cognitive categories, but not all categories [24]. The latter is also the conclusion of a systematic review of eHealth for patient engagement [25]. Moving beyond the psychology-oriented categorization, other patient engagement techniques, such as shared decision making and brief negotiated interviewing, have also been employed; in fact, interventions have been designed to integrate both techniques for engagement optimization [26]. Personalization has also been adopted in some studies as a patient engagement technique [16,27].

However, the main barrier in building a critical mass of literature on patient engagement with eHealth systems is the fact that very few studies address or report the topic. For instance, a systematic review on published trials discovered that only 23 of 2777 reviewed trials reported any patient engagement activities [28]. The literature review that was conducted in the framework of a different study focusing on older patients concluded that interventions to engage patients are generally scant and often poorly described [24]. In addition, in a recent systematic and mapping review on eHealth interventions employing electronic patient-reported outcomes (ePROs) for palliative cancer care conducted in the framework of the MyPal project [29], 17 of 24 eligible ePRO-based palliative care interventions for patients with cancer did not take patient engagement into account in the development or evaluation of the proposed digital health intervention. This may have been one of the reasons for the high attrition rates reported by the studies. Furthermore, four other studies recognized—mostly retrospectively—the need for patient engagement, and some attempted to measure it. Only 3 remaining studies actively attempted to optimize patient engagement during the design of the eHealth intervention or system.

#### Opportunities

In contrast to the other challenges discussed in this work, the solution to patient engagement may not be rooted in technology itself but rather related to the way technology is designed. To this end, there is a growing body of research demonstrating the value of cocreation and participatory design in the development of novel digital health services, including services for cancer care. Participatory design is one of the pillars of the revolutionary predictive, personalized, preventive, and participatory (P4) cancer medicine [30,31], and it requires the active involvement of users in the design or development phase of an eHealth system. Participatory design can provide a unique perspective on user acceptability, system usability, and the feasibility of the overall effort [32]. By involving a representative sample of a population of patients with cancer in the design or development of an eHealth system or service, one can eventually build an innovative solution that is expected to have good engagement with the target population.

The main barrier is a lack of research culture for considering the involvement of the end user (ie, in our case, the patient with cancer) in the design process. Despite this barrier, participatory design is expected to become the norm in eHealth technology development in the upcoming years.

#### Recommendations

The MyPal project (see the *Methods* section) is an example of a research effort that has committed to a *participatory design approach*, implemented primarily via a series of focus group discussions on eHealth interventions to be developed with the participation of all the involved stakeholders (patients as well as their informal carers and treating health care professionals) [33].

The use of the participatory approach as early as possible in the design of innovative technological solutions for patients with cancer presents a good opportunity to improve patient engagement [34]. The methodological tools, coming mostly from the field of qualitative research (eg, semistructured focus group discussions), are mature enough to support this design paradigm in eHealth, as these have been extensively validated in more generic software engineering environments. The employed participatory design process has driven the development of the MyPal platform for palliative cancer care and has provided a series of generalizable guidelines or recommendations for the successful application of participatory design processes for patients with cancer engagement, which are as follows:

1. Participatory design should start as early as possible in the development lifecycle of an eHealth system or service, and it should rely on established methodological tools.
2. Representative samples of the intended patient populations need to be selected for participation in the co-design and cocreation activities. This is especially important for heterogeneous patient populations.
3. Participatory design findings should be fused with other sources of knowledge (eg, a screening of unmet patient needs from cancer care in the scientific literature).

These recommendations can complement pre-existing published efforts to deliver more generic guidelines for developing engaging eHealth technologies. For example, the work presented in Karekla et al [14] specified 10 recommendations for researchers and clinicians interested in developing an engaging eHealth system. These recommendations are organized around 4 dimensions, namely a priori theoretical planning, human-computer interaction, tailoring and targeting to user groups, and active assessment of use. The eHealth technology development framework presented in Gemert-Pijnen et al [35] can also serve as a source of akin recommendations. Although the main goal of the framework is to improve eHealth technology uptake, some of the 6 working principles it introduces are applicable to the pursuit of patient engagement, especially the principle advocating for persuasive design techniques.

### Small Data Analytics

Data are fuel for any machine learning (ML) project [36]. It is well known that deep supervised learning algorithms are particularly data hungry—not only do they need a lot of data samples but the data also have to be manually annotated beforehand. However, big data sets with annotations (labeling, structuring, etc) are very rare, as proper annotations must be done by experts, and this is very expensive. Therefore, annotated data sets are mostly small data. Annotating data in a less costly manner remains to be a key challenge. A related challenge is to reduce the dependence of ML on annotation.

ML, especially deep learning, can effectively learn with big data. However, it cannot effectively learn with small data because of various issues, for example, overfitting, noise, outliers, and sampling bias, which can render the learned model effectively useless. Effective learning with small data is a challenge.

#### Opportunities

The annotation problem can be addressed via the use of annotation tools or services in many ways, such as (1) providing annotation tools so that annotation can be performed more effectively and easily, existing tools include Lionbridge artificial intelligence (AI) [37] and Computer Vision Annotation Tool [38]; (2) outsourcing the annotation task to an annotation service provider such as Amazon Mechanical Turk [39]; and (3) enabling an expert to teach their ML model while building and annotating their data set. However, none of these approaches solve the annotation problem scientifically. There are ways to deal with learning with small data problems, such as data augmentation, transfer learning, regularization, and visualization. However, these methods require skilled people, and their effectiveness is limited.

Another research question is regarding the procedure to add the value of ML results under the constraint of the available knowledge. Moreover, as knowledge and the latest clinical evidence, such as clinical practice guidelines (CPGs), are usually in paper-based formats and written in natural language, another research question arises regarding the procedure to automatically represent knowledge in a structured and computerized manner.

#### Recommendations

To solve the annotation problem scientifically, a desirable approach is to design a new ML algorithm that requires minimal feedback from human experts. This will only be possible if domain-specific constraints can be imposed on the learning process. This will reduce the model space as well as the variance in learning. Thus, one research question is on the procedure to reduce model space by domain-specific constraints.

An interesting approach to solve the *learning with small data* problem is to use domain knowledge in the learning process or *knowledge-based learning*. ML requires data as well as knowledge (common sense and domain-specific knowledge) implicitly or explicitly. When there are a lot of data, ML requires a small amount of knowledge; when there is not much data, ML requires a large amount of knowledge to reduce the model search space. Model-based ML can be seen as an example of knowledge-based learning, where knowledge can be specified by experts in the form of variables and their dependencies. This approach has been successfully demonstrated in various case studies. One weakness, however, is that formalizing knowledge is not a straightforward task, and it requires capturing experience from clinicians through inverse engineering and making clinical statements as explicit as possible for a computerized system. A desirable approach is a knowledge-based learning algorithm that (1) can be easily extended by data-driven findings and (2) uses standardized terminologies to provide interoperability and eases the updating and maintenance of the latest evidence. For example, within the DESIREE project, a digital breast cancer patient was formalized as a knowledge model ontology. The ontology employed standardized terminologies to identify univocally all identified clinical terms and procedures, which included the knowledge reported in standardized guidelines such as the National Comprehensive Cancer Network (NCCN) [40] or the European Society for Medical Oncology [41] guidelines in a computerized format, to be provided as recommendations through a decision support system (DSS) for multidisciplinary tumor boards during the decision-making process. Although formalization of knowledge through a model is almost a mandatory task in the very first steps of technical developments, it is a very time-consuming and costly task.

### Integration and Data Management

The multitude of successful instantiations of digital interventions has opened exciting new directions for acquiring, delivering, and sharing data, and has already proven the potential to leverage cost-effective, patient-centered cancer care applications [42-44]. Embedding patients into the iterative design process of digital intervention has been shown to enable developers to increase the relevance and effectiveness of the intervention [42]. Such treatments should be created with the acknowledgment of patients’ knowledge, attitudes, beliefs, preferences, and expectations of therapeutic outcome [45,46]. Thus, systems that support clinicians would benefit from a redesign that aligns cancer care more completely with patients’ needs and interests [47,48]. ML, through its pervasive impact [49], has the potential to provide a supportive tool for such a redesign task, given the powerful data understanding, generalization capabilities, and robustness [50].

#### Opportunities

Guided by the current state of the art, we identified a set of opportunities for digital interventions related to patient-centered cancer care. We have considered the most cited systematic reviews in the last 3 years (ie, to extract the innovation opportunities and technological limitations) and older systematic reviews (ie, studies from 2011 to tackle the initial adoption and strategies). These opportunities tackle the inherent challenges that we identified in practice. The first challenge is data collection and integration. Here, the core opportunity relates to exploring and exploiting the aggregation of heterogeneous and distributed data, including personal, professional, and health-related information [51]. Such systems are typically deployed as a platform to integrate retrospective, prospective, and day-to-day care data. However, data interpretation and translation remain to be an open challenge, whereas there is also a constant need for standard data formats such as Digital Imaging and Communications in Medicine and other standards applicable to electronic patient record or electronic health record (EHR) data. To address this, there is a clear opportunity to interpret patient information for the patient themselves by translating the clinical findings into an intuitive representation. This would ensure that the delivery of health information is done via a user-friendly interface.

When considering clinical data heterogeneity and the specificity of patient data, data projection is another challenge that is present in any technological intervention. We delineate a good opportunity to build individualized clinical recommendations based on the data interpretation projected in actions upon the patient. This initiative will be based on the patient’s risk profile and evidence-based guidelines (see the *Extracting Patient Portraits* section).

Finally, with regard to data sparsity, such clinical setups face the challenge of data completeness and augmentation. In contrast to clinical trial experience, data completeness improves with longitudinal care. This approach may be a solution to minimizing missing data of PROMs in research or clinical care settings in support of learning health care systems capable of augmenting the data [52]. To maintain the validity of the intervention, we believe that there is a good opportunity to consider and consolidate clinical data with the comprehension of lived experiences of patients, centered on patients [53].

#### Recommendations

On the basis of the identified challenges and opportunities, we established a set of recommendations for the systematic development of appropriate patient-centered digital interventions that ensure usefulness, adoption, and sustainability in cancer care [54,55]. This process can be extended with learning and generalization capabilities. ML algorithms excel at such tasks and constitute invaluable tools for any digital intervention.

Next, we map the challenges that we identified before practical recommendations. These recommendations can serve as a reference design for technological interventions when looking at integration and data management.

When addressing data collection and integration, we recommend embedding continuous user feedback and iterative prototyping in the intervention. This can be achieved by exploiting the multimodal nature of patient data (ie, personal, behavioral, professional, and health-related). ML techniques (ie, deep learning and hybrid neural networks) can fuse heterogeneous data in a common representation (ie, efficiently using very large data sets containing health care use data, clinical data, and data from personal devices and many other sources), as demonstrated by the recent deep learning systems used on multi-omics data sets to drive precision oncology care [56].

In terms of data interpretation and translation, we recommend the use of tools to extract and represent the medical substrate by synthesizing only relevant aspects in a declarative way. ML techniques (ie, deep learning—recurrent networks with word embeddings and distributed representations) can handle very large and sparse data (eg, device data may only be available for a small subset of individuals) to capture the sequential character of the data and are suitable for modeling context dependencies in inputs [57]. Such systems, which incorporate word embeddings encoding syntactic and polarity information in the language followed by deep neural network architectures, are already used to extract and normalize parameters within oncology care data.

To address the challenges in the area, we recommend the development of clinical projections (ie, mappings) from individualized patient recommendations to therapy plans that embed temporal, procedural, and reasoning processes. ML techniques, such as temporal hierarchical task networks, can dynamically generate personalized therapy plans for oncology patients [58], following a deliberative hierarchical planning process driven by the procedural knowledge described in oncology protocols [59]. Such instantiations use mappings to attach reasoning and procedural knowledge representation as well as their interpretation in a temporal planning process. The planning process allows us to obtain temporally annotated therapy plans that support decisions of oncologists. Moreover, such an ML technique offers the ability to deal with complex temporal and resource constraints, typical in cancer care.

Finally, tackling data augmentation, we recommend incorporating the lived experiences of the patients [60]. ML techniques, such as contextualized word embeddings, are suitable for improved text augmentation independent of any task-specific knowledge or rules and can process structured questionnaires for patients who, for instance, developed chemotherapy-induced peripheral neuropathy [61].

### Extracting Patient Portraits

Individual biomedical and nonbiomedical patient characteristics should guide any provided chronic care—digital or not. These insights are used to develop and validate *patient portraits* that can be employed in practice to determine optimal treatment strategies for subgroups of patients with similar cancer care needs and preferences. Building a *patient portrait* is hence an endeavor that follows a bottom-up approach that includes *patient profiling* based on patient phenotype algorithms [62-64]; intelligent patient profiling for the decision support of cancer treatment by exploiting clinical and genomic data [65]; personalized predictive modeling and risk factor identification using patient similarity [66]; and individualization beyond biomedical factors to also include demographic, socioeconomic, and psychological aspects [67]. To build a complete description of the multiple dimensions describing each patient, we identified a series of opportunities that describe all previously reviewed work.

#### Opportunities

The series of opportunities we identified target the selection of relevant heterogeneous and multimodal data correlated with the diagnosis. To extract the opportunities and frame our recommendations, we systematically analyzed a series of studies ranging from genomic data and phenotypes to demographics and psychological data. This methodology allowed us to capture the most relevant dimensions for extracting a patient profile. More precisely, the focus was on exploiting the correlations among multiple data sources to build a digital patient portrait consistent along all dimensions.

Such an initiative requires powerful data mining and ML algorithms, which can provide an efficient and compressed representation of a patient's digital profile, subsequently guiding therapeutic schemes.

One challenge identified relates to data relevance. Here, there is an opportunity to identify relevant genetic, phenotypical, physiological, lifestyle, and medical data correlated with the diagnosis. Exploiting such an opportunity can improve a patient's profile and the overall effect of the intervention, especially in progression-free survival [68]. Such studies demonstrate the feasibility of intelligent patient profiling that can select, within a clinically relevant time frame, a beneficial treatment for patients with no other treatment options.

Given the data deluge describing each patient, exploring and exploiting data correlations is another challenge we identified in the context of extracting a patient’s profile. This challenge provides a clear opportunity to exploit the correlations among the multiple identified modalities toward building an individual or personalized patient digital portrait that consistently captures all dimensions of the patient’s disease evolution.

Such an exploration unveils another challenge, namely multimodal data fusion [69], and the opportunity and potential that fusing extracted knowledge have toward a personalized therapeutic scheme [70]. Such systems, by combining data describing complementary perspectives on the same biological phenomena, can (1) separate correlated from discordant data, (2) extract the most informative features, and (3) estimate disease progression.

The last challenge we identified as being crucial in any effort to extract a patient’s profile is the possibility of embedding individualized data (eg, age, gender, ethnicity, health conditions, and social position) in patient cohorts [71]. Initial efforts were made to include family history for risk assessment and early detection of cancer; however, adherence to the study was low because of the limited technological support. This challenge brings along the opportunity to determine the population of interest and use this information in the process of *portrayal*.

#### Recommendations

On the basis of the identified opportunities and related challenges, we established a set of recommendations supporting a digital intervention design that (1) exploits available data, (2) extracts underlying correlations, and (3) integrates the multitude of representations in a structured object guiding therapeutic interventions in cancer care.

When addressing the challenge of identifying data relevance, we recommend selecting the data or feature subset that best characterizes the statistical property of a certain variable (ie, a certain patient) subject to the constraint that these data or features are as mutually dissimilar to each other as possible but as marginally similar to a certain class of patients as possible. For this task, ML tools such as minimum redundancy feature selection (ie, Minimum Redundancy Maximum Relevance) [72]) can be used to accurately identify the characteristics of patient features or data and narrow down their relevance. Such techniques provide an integrative approach to patient-centered data and demonstrate the potential of feature selection in data analysis and predictive patient-specific outcomes [73].

To address data correlations, we recommend the use of ML models, such as long short-term memory (LSTM) [74] networks, which can be used for their ability to effectively model varying length time series data and capture long-term dependencies and correlations. From modeling the patient life expectancy from medical records [75] to complete patient trajectories [76], modeling the disease trajectory and care processes, assumes mining electronic medical records that are episodic and irregular in time. Such models capture long-term temporal dependencies and are well suited to modeling clinical data because the evidence of certain conditions may be spread apart over several hours or days, and important symptoms may present early on in a patient’s trajectory.

The next challenge identified is the fusion of the available multimodal data. Our recommendation is to use a data-driven feature learning class of approaches. Typically, they are based on deep networks that can directly learn the hidden characteristics of the data from different sources. As such, we recommend, for instance, the use of deep neural networks to extract features from genomic and clinical data [77], convolutional neural networks to extract features from pathology images [78], and recurrent neural networks for text and medical records data [79].

Finally, the last identified challenge is the possibility of embedding individualization data in the patient profile. We recommend performing individual cognitive interviews and focus groups with patients to learn about their relevant needs, experiences, fears, aspirations, and expectations.

From the ML point of view, a solution for developing personalized patient embeddings that is capable of processing such data is a combination of well-proven autoencoder methods with extensions to some of the metrics to account for data sparsity and multimodality [80]. Such studies also provide a methodology describing how these networks can be designed, built, and applied to tasks of integrative analyses of heterogeneous cancer data.

A patient portrait that can capture complex relationships in physiological signals, nonbiomedical data, and personal data embedding is key to accurately predict the stages of interventions for different patients and is necessary for successful personalized therapy.

### Learning Patient Disease Trajectory for Personalized Diagnosis

Cancer is remarkably heterogeneous across individuals. This heterogeneity makes treatment difficult for caregivers because they cannot accurately predict how the disease will progress to guide treatment decisions. Therefore, tools that help to predict the individualized trajectory of cancer can help improve the quality of health care [81,82]. Given the assessment of the current state of the patient (ie, patient profile, physiology, neuroimaging, blood biomarkers, and physiologic testing) along with the therapeutic scheme, a digital intervention would use ML or predictive systems to infer disease evolution or remission to be able to guide subsequent therapy scheme planning [83]. Such a trajectory can also support the detection of behavior change in patients [84].

#### Opportunities

A significant need relates to making disease predictions by leveraging baseline information and additional time-dependent clinical markers as they are collected. Such an approach is the focal challenge of personalized medicine: integrative analysis of heterogeneous data from an individual’s medical history to improve cancer care. We identified several key challenges and associated opportunities linked to this.

The first challenge relates to the fact that markers in clinical data are irregularly and sparsely sampled. Here, we identified a valuable opportunity for handling data and choosing specific latent variable models to summarize and extract information from the irregularly sampled and sparse data. This should simultaneously ensure sidestepping the issue of jointly modeling the data-generating processes [85]. Such systems should build a temporal representation of care trajectories in the form of a time-ordered state sequence. Moreover, in addition to the routine identification of key dates and events in patients’ care trajectories, such systems should identify initially fragmented data across numerous sources.

Another challenge identified relates to the learning of a disease trajectory and is linked to the inherent computational complexity. Imposed by the clinical setup, we identified an opportunity to predict the entire disease progression trajectory from the observed patient records without many training labels on the ground-truth stages that a patient acquired, in mechanistic models of disease progression. A joint approach is prone to alleviate the inherent variability in prediction.

This challenge opens the stage for the next challenging point, namely continuous adaptation and updates in the face of disease progression heterogeneity. In handling such a challenge, there is a clear opportunity for continuous-time adaptation and updates to new observations and new data (markers). This provides novel computational methods for predicting, for instance, disease phenotype from molecular and genetic profiling [86].

Finally, another challenge we identified refers to the observed versus latent data artifacts. Addressing such a challenge demands tools for capturing latent factors in disease expression and not only observed features as a crucial aspect for patient-tailored cancer therapies. We further elaborate this in the following *Recommendations* section.

#### Recommendations

In this section, we match the challenges to practical recommendations in designing digital interventions when predicting disease trajectories for patients with cancer.

To handle irregularly and sparsely sampled markers in clinical data, we recommend, from the ML perspective, the use of discriminative models that condition on marker histories instead of jointly modeling them. Such an approach will not be sensitive to miss-specified dependencies across marker types and inherent irregularities and sparsity. For example, functional data analysis [87] can be employed for sequences of measurements that are assumed to be samples from an underlying continuous function. However, coefficient estimates can have a high variance in time series.

However, the task of predicting the disease trajectory comes with its inherent computational complexity. To address this challenge, we believe that an ideal candidate would be a machine model that grows linearly in the number of marker types included in the model. This makes such a task applicable to cancer prognosis, where many different markers are recorded over time. Generative models can account for disease trajectory shapes using components at the population, subpopulation, and individual levels, which simultaneously allows for heterogeneity across and within individuals and enables statistical strength to be shared across observations at different *resolutions* of the data [88]. Moreover, such systems can learn accurate and interpretable structured representations for disease trajectories by adapting their attention weights that determine the dependence of future disease states on past medical history.

Independent of the prediction model, the challenge is to continuously adapt and update in the face of progression heterogeneity. From the ML point of view, we recommend the use of a model capable of being applied dynamically in continuous time and updated as soon as any new data are available (eg, hidden Markov models). Such approaches can model the transition of disease stages or states, which implies that the progression is continuous, and the transition probability to the future state relies only on the current state and the time span. Instantiation of such causality-based ML was used to infer the underlying somatic staging of tumors from next-generation sequencing data [89].

Moving away from the modeling decision, the last challenge lies in the observed versus latent data artifacts. We believe that a powerful tool is an ML model that accounts for latent factors and covariates influencing disease expression, as standard regression models rely on observed features alone to explain variability [90]. For example, LSTM models over physiological word inputs from health records significantly improve performance as their representation encodes important information about what is *normal* for each physiological value or is more robust to sparseness in the physiological data.

### Technological Interventions in Cancer Rehabilitation

Over the past decades, early diagnosis, new drugs, and more personalized treatment have led to impressive increases in survival rates of patients with cancer. However, the most mitigating side effects of commonly used therapies are a severe problem in oncology, leading to dose reduction, treatment delay, or discontinuation [91,92].

#### Opportunities

With the increasing number of cancer survivors, more attention is being paid to persistent sequelae of tumor treatment and supportive measures [93-95] used as adjuncts to mainstream cancer care to control symptoms and enhance well-being [96-100]. The broad literature overview allowed us to identify a series of challenges and the associated opportunities that digital interventions could offer in supportive care.

The first challenge we identified is the identification of therapeutic sequelae. This challenge offers the opportunity to develop interventions capable of assessing what deficits (sensory, motor, and/or cognitive) a specific patient has as a consequence of cancer therapy.

The next challenge arises when the intervention needs to quantify the magnitude of therapy sequelae. Here, we identify a clear opportunity to measure the level of deficit or dysfunction induced by the therapy. This is crucial in (1) designing the follow-up therapy scheme, (2) choosing a rehabilitation strategy, and (3) determining the therapy trajectory and dosage.

The last challenge we identified is the adaptive parametrization of rehabilitation. This challenge brings a valuable opportunity to take steps toward personalized treatment, namely, to parameterize the rehabilitation scheme according to the specific deficit type and level to drive rehabilitation.

#### Recommendations

To cope with patient sensory, motor, and cognitive deficit variability, it is necessary to perform a precise assessment of the 3 different dimensions. We believe that the 3 main challenges we identified as high-potential opportunities for digital interventions are also good candidates for ML algorithms. This technology can learn underlying correlations in patient data and generalize for robust prediction [101]. The mapping from the challenge to the recommendation is provided in the remainder of this section.

When tackling the identification of therapy sequelae, we recommend exploiting and mining large sets of structured and unstructured data describing a patient to identify correlations among various data types and how they map to a certain type of dysfunction. We propose the use of semisupervised techniques (eg, transductive support-vector machines [102]) that will only require a limited number of labels to generalize well.

Moreover, to address the magnitude of the therapy sequelae, we recommend the use of deep learning models, especially convolutional neural networks. This is because such networks are capable of learning relevant feature representation from unstructured data, such as pathological images or medical records. This, subsequently, allows deep learning methods to achieve good results in tasks such as regression, detection, and segmentation, which underlie the magnitude estimation.

Finally, addressing the challenge of adaptive parametrization of rehabilitation, we recommend using methods that combine learning capabilities for regressing arbitrary nonlinear functions (ie, deep learning—encoding the type of deficit covariance with the magnitude) and adaptation through guided searches in parameter spaces (ie, reinforcement learning—finding the best parameters of the rehabilitation scheme—dosage, type, and length that best fit the regressed function).

### Addressing Current Interoperability Challenges

Patient data needed for the provision of the best treatment are often scattered across different systems rather than stored in a single location. Technologies that facilitate care coordination through interoperability are improving, but a seamless flow of information from one care setting to another still requires more progress. Without having a full picture, it is difficult to provide the best care in an era where cancer is considered to be a chronic disease, and there is an increased demand for consistent follow-up in terms of monitoring and early management of symptoms that indicate that cancer might have returned.

Interoperability is a primary consideration to achieve communication among applications, medical devices, and health care providers [103], although the growing demand for secondary use of clinical and administrative data increases the pressure to solve relevant challenges [104].

The currently popular use of EHRs has alleviated some of the barriers in using data from medical records for research, although fully interoperable electronic medical record systems are not yet a reality. Several efforts to develop and apply standards in the collection, extraction, and integration of data by standardization bodies, governments, the research community, and industry are in progress [105] with the aim to establish and adopt clinically relevant, integrated standards covering the entire oncology sector. Organizations such as Health Level Seven International [106] and Personal Connected Health Alliance Continua [107] help to deliver standards-based, open specifications that can support the flow of data from the point of capture into EHRs in the same format and coded content. Cancer-specific apps that exploit platforms and interoperability standards, such as SMART [108] and Fast Healthcare Interoperability Resources [109], are emerging [110-112].

Standards, such as those developed in the United States and Canada, to guide EHR vendors and public health central cancer registries in the implementation of standardized electronic reporting [113-115] can be used with third-party terminologies.

It is a fact that some data resolution may be lost during the process of mapping EHR fields to a formally described abstraction layer, which may be alleviated by the use of knowledge models such as ontologies as a knowledge background mapper; however, these common interfaces support queries across EHRs or the extraction of patient data in the same format to allow the merging of patient sets between numerous institutions.

#### Opportunities

Health information technology (IT) brings clinical data and patient information together and guides oncologists in making evidence-based care decisions that lead to improved outcomes. The potential benefits of interoperable interconnected tools and health systems are particularly important for oncology [116], as providing cancer-wide care depends on access to accurate and complete information as well as extensive coordination among patients, caregivers, and diverse provider groups through treatment and survivorship. Connecting the EHR has the potential to support diagnosis assistance for complex patients [117].

Data must be able to provide a complete look at the patient’s medical history so that physicians can see what cancer medicines and treatments did or did not work. Clinicians also need to be able to avoid recommending the same procedure twice, prescribing a medicine the patient already tried, or missing results from a diagnostic test.

The need for consolidation and standardization efforts to create interoperable solutions [105] and the need for the cancer informatics community for national initiatives for data standardization and large-scale multidisciplinary research collaborations are timely and critical. In addition to supporting cancer care, cancer-related standards will help improve surveillance and research. Similar to migration from paper to electronic records, the shift toward data interoperability between EHR vendors may require policy changes [118,119].

#### Recommendations

As already proposed by [120], enabling interoperability among institutions and individuals that support care delivery across the cancer continuum is considered essential. Doing so requires developing, testing, disseminating, and adopting technical standards for information related to cancer care across the continuum to optimize the flow of information to serve the needs of caregivers, patients, and providers. To achieve this, standard open application programming interface platforms should be developed and used to facilitate the development of cancer-related apps.

As mentioned in [121], standards and protocols that aim to enhance the interoperability of different data sets are a highly relevant field for policy action. Incentives for the promotion of standards for the interoperability of clinical data, such as EHR or genome experiment data, should be developed, promoted, and incentivized to allow for the pooling of data, and comparison of system-level research should be provided for the comparison of research outcomes.

The open use and sharing of big data, without compromising patients’ rights to privacy and confidentiality, should be promoted.

### Patient-Clinician Shared Decision-Making Processes

Current clinical care is gradually moving toward patient-clinician shared decision making, as patient involvement can provide insights into best health states or outcomes in each case, apart from establishing a partnership that will help clinicians understand their patients’ preferences [122]. In a complex disease, such as cancer, making a clinical decision is a difficult task because of the trade-off between the level of treatment benefits and the impact that adverse events or symptoms could have on the patient’s life. This is especially crucial in cases where the expected medical outcomes are similar for different clinical procedures (ie, also referred to as *equipoise*), requiring an individualized and personalized health care process involving the clinician and the patient in a close interaction for the best decision making [123]. Nevertheless, actual CPGs still do not include or barely mention the impact of patient preferences along with clinical evidence.

#### Opportunities

It is necessary to overcome many barriers to ensure success in the inclusion of patient preferences during the decision-making process and to track their impact in the care services provided [124]. First, patient preferences should be considered as population knowledge that follows some general trends and not just as individual one-off cases or subjective and variable factors. This would allow including them along with the clinical evidence reported in the studies to identify *preference-sensitive* decisions (eg, decisions having lifelong implications or an uncertain benefit to the patient, unclear or conflicting evidence, risk of having side effects that negatively affect the patient’s quality of life) of high levels of uncertainty about the best clinical procedure to follow [125].

#### Recommendations

As a complex disease, cancer involves many clinical specialties, with the need of creating a clear taxonomy (ie, systematic categorization) for patients’ preferences that will serve as a standardization over all involved disciplines (eg, analysts, clinical psychologists). This kind of approach would help in the harmonization of the different points of view on the measurement of patients’ preferences by labeling and extracting this information in a processable and understandable way [126,127]. Finally, with regard to the previous points, building a methodology to synthesize the current knowledge on preference trends to be able to describe preference-based decisions along with clinical-based evidence would have a great impact on health care performance, as it has been proven that preferences strongly influence the decision-making process [128,129].

### Assessment of Clinical Evidence-Based Recommendations, Including PROMs

As presented in the previous sections, the evidence used in medical practice is based on clinical guidelines, which are often used as the evidence basis for the clinical DSS (CDSS) [130]. These guidelines are developed by multidisciplinary groups and are based on a systematic review of the scientific evidence, and their recommendations are explicitly linked to the supporting evidence and graded according to the strength of that evidence [131]. As reported by Harbor et al [131], the levels of evidence of the recommendations are based on study design and the methodological quality of individual studies, which is called the quality of evidence. Therefore, the latest approach also considers the strength of the recommendations, which is a trade-off between benefits and harms of a treatment, considering the quality of evidence [131,132]. Several oncology guidelines, such as the NCCN CPGs in Oncology [133] applied in DESIREE, provide quality of evidence measure, and it is up to the clinician to consider the strength of the recommendation depending on the specific patient situation. However, the subjective measures of patients with cancer, such as fatigue affected by oncological treatments, have not been systematically considered during the decision-making process. Making this information available during the decision-making process also provides other relevant and real-world quality measures to the treatment recommendation to best support clinicians and patients in their treatment decisions.

#### Opportunities

CDSSs provide the opportunity not only to quickly access the latest available evidence but also to incorporate new sources of information that can support the decision-making process. At the same time, the new IT era enables the acquisition of PROMs using questionnaires or even more sophisticated wearables that can measure activity, sleep, or other vital signs that can be translated to patient outcomes. These automatic ubiquitous technologies increase the knowledge required by patients. Processing these data and deducing the desired results will be very helpful. For example, tracking the daily activities of a user provides estimates of how active a patient is, which can be correlated with their depression and/or fatigue levels (variables that are gathered using questionnaires such as the European Platform for Cancer Research—Quality of Life Questionnaire with 30 items). Other techniques could also aid the acquisition of patient outcome information, such as the Ecological Momentary Assessment tests (short questionnaires that are sent to the patient frequently to obtain updates on their status). Thus, the collected data could be used to assess how good the treatment given was for each patient, not in the scope of a randomized control trial but in the real-world environment, without having a specific patient population, but the whole population.

#### Recommendations

In this context, in the DESIREE project focusing on primary breast cancer, new ways of including PROMs to assess guideline recommendations were explored. The aim of including PROMs does not conflict with the quality of evidence and strength of recommendation measurements but provides other quality attributes that contribute to the decision-making process, considering the patient status reported by the patients themselves.

There are some specifications that should be considered for this quality assessment, which could be named questionnaire based–patient-reported outcome measures, and they include (1) consideration of standard questionnaires for each medical case, such as the International Consortium for Health Outcomes Measurement questionnaires [134], (2) definition of specific protocols to assess the results of the questionnaires, and (3) determination of how these results should be used during the decision-making process.

### Ambiguity on Clinical Guidelines Used for Clinical Decision Support

When implementing CPGs, several characteristics must be considered to ensure both good health care quality levels and clinicians’ satisfaction. They must assure the validity and reliability of their clinical content, along with their clinical applicability in real clinical settings, and must be clear when defining the procedures to be followed in the current clinical performance procedures within a health care system [135,136]. Nevertheless, several barriers cause the dissemination of the guidelines to be tedious and difficult, mainly because of the ambiguity of the knowledge defined in them. The lack of awareness and familiarity with the recommendations provided in the guideline, a lack of agreement due to different clinical interpretations and simplification of the clinical knowledge reported in the guidelines, and the lack of outcome expectancy are some of the reported barriers that cause a lower adherence of clinicians to guidelines and have an effect on their implementation, compliance, and adherence in real clinical settings [137,138].

#### Opportunities

Actual trends move toward highly interactive computerized systems focusing on intuitively presenting complex clinical cases, where clinicians may access and check computerized clinical data and take away insights from all of this information in a more natural and intuitive way, alleviating the ambiguities of the guidelines through a data-driven approach guided by previous practice [139]. The digital implementation of the CPGs provides evidence-based decision support (ie, computer-interpretable guidelines [CIGs]) [140]. To achieve this, the knowledge available has to be formalized in a manner that is correct and of good quality, by following a consistent and adequate methodological workflow of the clinical processes and objectives, trying to reduce or provide some solutions to the paper-based guidelines ambiguities [141].

#### Recommendations

The proposed directions for realizing these needs include the promotion of standardized clinical terminology that facilitates the understanding and univocal interpretation of the clinical data to be analyzed and the clinical knowledge formalized in the CIGs (eg, the Breast Imaging Reporting and Database System standard for breast anomalies [142]); to be effective, clinical guidelines need to be integrated with the care flow and should provide patient-specific advice when and where needed. Hence, their formalization as CIGs should make it possible to develop CIG-based DSSs, which have a better chance of affecting clinician behavior than narrative guidelines. This will help in providing optimal personalized guideline-based recommendations, avoiding ambiguities, and at a reasonable cost and implementation effort [143].

### Up-to-Date Clinical Evidence Guidelines for CDSS

Clinical guidelines are tedious to develop, and it is even more difficult to interpret and put them into a computer-interpretable way. This usually requires the close collaboration of knowledge engineers and medical experts. A closely related and important issue is the guideline development process or how CPG development working groups are composed. Usually, these teams comprise quality auditors or managers who are guided by their opinions, interests, and experience and intend to formalize evidence, seeking appropriateness of the provided recommendations but ignore the iterative and causal reasoning of clinicians [144]. Depending on the clinical context and according to the approaches followed for developing, disseminating, and implementing them in practice, CPGs can be more or less successful when reporting the latest clinical evidence [145]. Thus, the rapid advances in clinical practice have made the task of updating the guidelines used for the CDSS more challenging. In particular, in oncology, the number of discoveries is increasing rapidly. As reported by Beatty [146], drug label information and indications do not always keep up with meaningful advances in oncology resources. However, clinicians may be aware of the potential benefits of a particular therapy. Hence, Beatty [146] reports that continuous vigilance (in the form of continuing medical education and literature review) is a survival attribute for medical oncologists. Consequently, there is also a challenge to update the CIGs and have up-to-date guideline-based CDSS.

#### Opportunities

In this context, it is crucial to have tools supporting the easy updating of CIGs used for CDSS by clinicians themselves or knowledge engineers who do not necessarily have to understand the technology and/or the programming language used. Thus, interfaces that are easy to use and easy to understand are required for this purpose. This limitation was highlighted in the DESIREE project; however, this is an issue not only for cancer care but also for other clinical specialties.

#### Recommendations

Therefore, we propose an authoring tool for CPG formalization [147] that should at least fulfill the following requirements: (1) enable the input of guidelines’ information in an easy and visual manner, such as the form of rules or flowcharts, and (2) enable the modification of CIGs previously formalized in the system. In addition, future work should focus on providing a tool for detecting modifications in guidelines as well as semiautomate the formalization of guidelines, using natural language processing.

### Trust and Reliance on Cancer Care

The change in both hardware and software over the past decade has been remarkable. Equally noteworthy has been the ever-increasing internet speed and the accompanying growth in the demand for connectivity. This growth and development have increased our ability to take on challenging projects to improve early diagnosis and improve the quality of cancer care. However, most discussions relating to health data and associated analytical tools often emphasize data privacy and security at the expense of other topics. These talks often overlook the dynamic nature of both health data and the software used in the analysis of those data. In addition, the popularity of internet-based apps and the use of such tools by patients for self-diagnosis necessitate a call to action. There is a need to examine the reliability and trustworthiness of health-related computational tools used in diagnosis and DSS by health care providers. Here, we will focus on the reliance and reliability of computational tools with an emphasis on cancer care and diagnosis.

#### Opportunities

Reliance and reliability of information in health care are incredibly important. These attributes are particular when it comes to genetic information associated with cancer, which is highly critical in the development of optimal clinical intervention strategies. With an increasing number of people falling victims to medical misinformation and propaganda on the internet [148], advocacy to develop reporting and assessment standards has been long overdue. The internet undoubtedly provides patients with countless sources of health information related to cancer diagnosis and care, some of which represent the gold standard, whereas other sources remain to be of ill repute. The internet promotes decentralization and web democratization of access. Having an open-source or access model certainly helps to promote this agenda. These models provide help and are available to many without the control of smaller powerful broker or agents. However, it challenges the core process of software development, which includes specification, design, development, verification, validation, and management. Although inspection or peer review is the method of choice to check for static processes and software testing a true validation and verification method to check the system, the former fails to check emergent properties such as reliability and performance.

There is no turning back from this path of dependence on the tools and information available via the internet. People have a reasonable expectation of establishing trust and validation of these tools. Initiatives such as the Secure Socket Layer certification system, implemented for encrypting sensitive information sent across the internet or the use of digital badge to indicate or attest to adhere to an acceptable standard and/or individual skill competency (*seal of approval* from prominent institutions) or the use of the message-digest algorithm 5 (MD5) hash to ensure data integrity work to build and maintain that trust. Health IT requires a method to assure the credibility of the results generated by various computational tools available on the web. Unfortunately, none of the previously listed solutions are applicable to health software because of the dynamic nature of data as well as the software (as many deviate from the original specifications for which acceptable test and peer review of the result exist).

#### Recommendations

Having reliable information alone is not sufficient for people to construct their foundation of trust. People may have a distrust on the reliability of official information on certain topics because of previous unpleasant experiences. One conclusion that can be drawn from our experience in developing relevant computational tools over the past decade is the questionable credibility of the results obtained from such tools. A review of the literature shows a great deal of volatility in the availability of health IT resources and shows that providing explanations for software errors is an acceptable approach. Building on this notion, a certificate that discloses who is responsible and what tests are done or can be done to validate or test the trustworthiness of the output, something along the line of the MD5 checksum, could be envisioned as an acceptable solution to address this issue. Such a certificate could include the versions of the data and the software in a report to help explain the deviation from the previous version. This could be seen as a reasonable step in the right direction before the availability of peer-approved permanent solutions.

### Trust in Computer-Aided Diagnosis Systems

An important issue emerging for decision support in medical diagnosis is the trust that clinicians might have in the outputs generated by a computer-aided diagnostic system. This is a highly relevant issue for tissue characterization in general and image-based tumor classification in particular. Hence, it should be considered whether the metrics such as accuracy (ACC) and area under the curve (AUC) correlate well with confidence in the algorithms used in computer-aided design (CAD) systems. If the CAD system provides a recommendation with moderate or low confidence, then a radiologist may deem the recommendation to be useless, even if the classifier being used has a high overall value for ACC and/or AUC. However, if the confidence is much higher, the clinician may deem the recommendation more useful in supporting their decision making. Therefore, building CAD systems in which clinicians have confidence is essential if those CAD systems are to be adopted and are to play a role in fully exploiting the range of digital information available for assisting diagnosis.

Recent studies discuss the failure of CAD systems in terms of a lack of *trust* by clinicians in the outputs that the CAD systems generate [149-151]. When designing and evaluating an algorithm within a CAD system, technical developers tend to report metrics such as ACC and AUC to demonstrate the performance of a classification method. However, such metrics do not measure the degree of confidence in individual recommendations made by the CAD system. For example, a CAD system may produce a high AUC value very close to the optimal value of 1, but most of the cases might be classified with low confidence (as measured by the likelihood or probability value associated with the recommended class). Indeed, studies have shown that the use of CAD systems that incorporate inadequate metrics can be detrimental to diagnosis [152].

#### Opportunities

Exploratory studies for breast mass classification [153] and classification of microcalcifications in mammograms [154] using data sets taken from the Curated Breast Imaging Subset of DDSM [155] showed that although most classifiers produce similar overall ACC and AUC values, their performances differ significantly in terms of confidence measure. High ACC or AUC does not provide a full indication of the confidence level of a CAD system. This aspect of the usability of CAD methods is a key but overlooked challenge. Most cancer CAD systems have used ACC and AUC as the main evaluation metrics; however, the probability outputs of the classifier must also be considered and harnessed to measure the degree of certainty of the system in its decision making. Hence, if clinicians are to have confidence in the support provided by a CAD system, the classifier that is chosen to be implemented as part of the system should not necessarily be the one that performs best overall using the standard metrics. CAD support systems must embody reliable confidence measures as one of their key elements [156]. It is therefore essential that the domain of trust in CAD systems should be explored to incorporate trust into the initial classifier design when such algorithms are to be embedded into a cancer CAD system.

#### Recommendations

To fully exploit the range of digital information available for assisting diagnosis, it is important to identify and implement specific actions to increase the trust of physicians in cancer CAD systems and overcome the barriers to adoption of such systems. Most cancer CAD systems have used ACC and AUC as the main performance evaluation metrics; however, confidence measures must also be considered, as the traditionally used metrics are inadequate for informing clinicians in terms of the confidence that they might place on the recommendations provided. Besides, research in the design of classifiers that are incorporated into CAD systems is essential if future CAD systems are to be trusted by clinicians and adopted as a valued and reliable, and indeed routine, element of the cancer diagnosis process.

### Regulatory Roadmap for Validating the Effectiveness of AI–Based Models for Clinical Decision Making

Data and image analysis algorithms usually require regulatory approval by the Food and Drug Administration (FDA) in the United States and conformity assessment leading to a Conformité Européenne mark in the EU [157], under the new Medical Device Regulation. Both EU and US regulations classify medical devices according to risk classes, and those that fall under certain risk classes are required to undergo clinical trials to be marketed [158]. To date, these guidelines target systems with so called *locked* algorithms, that is, algorithms whose functions do not change, rather than *adaptive* ones, that is, whose behavior can change over time based on new data [159]. Adaptive AI–based tools have the potential to autonomously “adapt and optimize device performance in real-time to continuously improve healthcare for patients.” The FDA has already approved several AI- or ML-based software as a medical device (SaMD), but these have only included algorithms that are *locked* before marketing [160].

#### Opportunities

Regulations that aim to validate AI in a safe and transparent way must consider doing so without compromising the potential of AI. Moreover, characteristics of AI that may be seen as risks such as biases in data, its *blackbox nature*, and model degradation, among others, have to be included in the validation process to better ensure safety and build public trust [161]. The FDA recently proposed an approach that aimed to “have tailored, pragmatic, and least burdensome regulatory oversight” while validating the continued safety, effectiveness, and performance of SaMDs. The framework recommends (1) establishing clear expectations of quality systems and good ML practices, for example, demonstrating analytical and clinical validation, (2) creating SaMD prespecification sheets and algorithm change protocols to qualify for a premarket review on the safety and effectiveness of the AI-based tool, (3) preparing an approach for *AI- or ML-based software modifications* (eg, data for retraining), and (4) implementing mechanisms that support *transparency and real-world performance monitoring*.

In the EU, there are no regulatory guidelines that specifically cover AI in health care. Nevertheless, draft ethics guidelines have been published by the High-Level Expert Group on AI in April 2019, proposing 7 key requirements that AI systems should meet to be realized as *trustworthy AI*: human agency and oversight, including fundamental rights; technical robustness and safety; privacy and data governance; transparency, diversity, nondiscrimination, and fairness [162,163]; societal and environmental well-being; and accountability. The guidelines also propose technical and nontechnical methods to implement the 7 key requirements and recommend that these should be continuously evaluated and addressed throughout the AI system’s life cycle.

#### Recommendations

The frameworks enumerated above are, however, still being piloted or discussed. To properly define a regulatory framework for users, stakeholders and use cases (data flows) should be identified and defined. New regulatory frameworks should be built to provide guidance for the validation or qualification of AI tools within different scenarios and pathways (for nonclinical or preclinical use or clinical use), taking into consideration the adaptive nature of AI-based tools. The framework should consolidate input from scientific experts and health authorities and should take into consideration published relevant guidelines, for example, from the High-Level Expert Group on AI, the Medical Device Coordination Group and relevant implementing EU legislation, and international regulations (eg, from the FDA).

Table 1 summarizes the various topics discussed, the related works for each topic, the opportunities, and the recommendations from the workshops.

**Table 1:** Topics, opportunities, and recommendation in cancer care.

Topic or section (references)	Opportunities	Recommendations
Patient engagement and participatory design [11-29]	Involvement of real users Identification of user needs Unique perspective on user acceptability, usability, and feasibility	Participatory design approach early and throughout the design process Focus groups with stakeholder representatives Fuse findings with those from other sources
Small data analytics [36]	Address the annotation problem via appropriate tools Enable experts to teach ML^a^ models that automatically build and annotate their data sets Automatically represent knowledge in a structured and computerized way	Design new machine learning algorithms that needs minimal feedback from human experts Use knowledge-based learning that can be extended by data-driven findings easily and that uses standardized terminologies to provide interoperability and ease the updating and maintenance of the latest evidence
Integration and data management [42-50]	Exploiting aggregated, heterogeneous, and distributed data Translating the clinical findings into an intuitive representation for the patient Building individualized clinical recommendations based on the data interpretation projected in actions upon the patient Data completeness and augmentation	Embed continuous user feedback and iterative prototyping in the intervention Usage of tools to extract and represent the medical substrate by synthesizing only relevant aspects in a declarative way Development of clinical projections from individualized patient recommendations to therapy plans that embed temporal, procedural, and reasoning processes Incorporation of lived experiences of the patients
Extracting patient’s portrait [62-67]	Exploit correlations among multiple data sources to extract patient profile Use data mining and machine learning to guide therapeutic schemes Identify relevant genetic, phenotypical, physiological, lifestyle, and medical data correlations with diagnosis Provide an integrative approach to patient-centered data and demonstrate the potential of feature selection in data analysis and predictive patient-specific outcomes	Exploit available data Extract underlying correlations Integrate the multitude of representations in a structured object guiding therapeutic interventions in cancer care
Learning patient disease trajectory for personalized diagnosis [81-84]	Handling data and choosing specific latent variable models to summarize and extract information from the irregularly sampled and sparse data Learning of a disease trajectory is linked to the inherent computational complexity Continuous adaptation and update in face of disease progression heterogeneity Observed versus latent data artifacts	Use of discriminative models that exploit conditions on marker histories instead of jointly modeling them Focus on machine models which grow linearly in the number of marker types included in those models Use of a model capable of being applied dynamically in continuous time and updated Exploit models that account for latent factors and covariates influencing disease expression
Technological interventions in cancer rehabilitation [91-102]	Cope with patient sensory, motor, and cognitive deficit variability Identify therapy sequelae	Perform a precise assessment of patient’s sensory or motor or cognitive deficit variability Use machine learning algorithms to identify underlying correlations in patient data and generalize for robust prediction Exploit and mine large sets of structured and unstructured data to identify correlations and map to a certain type of dysfunction
Addressing current interoperability challenges [103-111,113-115]	Provide cancer-wide care Support diagnosis assistance for complex patients Provide a complete look at the patient’s medical history so physicians can see ineffective treatments Improve surveillance and research	Develop, test, disseminate, and adopt technical standards for information related to cancer care across the continuum Optimize the flow of information to serve the needs of caregivers, patients, and providers Develop and use standard, open application programming interfaces Promote incentives for the pooling of data and comparison of system-level research Support open use and sharing of big data, without compromising patients’ rights to privacy and confidentiality
Patient-clinician shared decision-making processes [122-129]	Inclusion of patient preferences during the decision making and tracking of their impact in the provided care services Identification of *preference-sensitive* decisions	Create a clear taxonomy (ie, systematic categorization) for patients’ preferences to serve as a standardization Harmonize different points of view to facilitate labeling and extraction of information in a processable and understandable way Build a methodology to synthesize knowledge
Assessment of clinical evidence-based recommendations, including PROMs^b^ [130-133]	Quick access to latest available evidence Incorporate new sources of information that can support the decision-making process Increase the knowledge required by patients Effectively assess how good the treatment given was for each patient, not in the scope of a randomized control trial, but in the real-world environment	Explore new ways of including PROMs to assess guideline recommendations Exploit PROMs in the decision-making process, considering patient status reported by the patients themselves Consider and use existing quality assessment specifications for PROMs
Ambiguity on clinical guidelines used for clinical decision support [135-138]	Insight from complex clinical cases in a natural and intuitive way Patient-specific advice when and where needed	Promotion of standardized clinical terminology Integration of clinical guidelines with care flow
Up-to-date clinical evidence guidelines for CDSS^c^ [144-146]	Create tools that support the easy updating of CIGs^d^ for clinicians Interfaces that are easy to use and understand are required for this purpose for CIGs	Generate tools that enable the input of guideline information in an easy and visual manner and enable the modification of CIGs previously formalized in the system Provide a tool for detecting modifications on guidelines Semiautomate the formalization of guidelines using natural language processing
Trust and reliance on cancer care [148]	Assurance of the credibility of results generated by various computational tools available on the web	Provision of a certificate that discloses who is responsible and what tests are done or can be done to validate or test the trustworthiness of the output Include the versions of the data and the software in a report to help explain the deviation from the previous version
Trust in computer-aided diagnosis systems [149-152]	Increase confidence in the support provided by a CAD^e^ system	CAD support systems must embody reliable confidence measures as one of their key elements Incorporate trust into the initial classifier design when such algorithms are to be embedded into a cancer CAD system
Regulatory roadmap for validating the effectiveness of AI^f^-based models for clinical decision making [157-160]	Validation of AI in a safe and transparent way without compromising the potential of AI	Identify and define users, stakeholders, and use cases (data flows) Build regulatory frameworks aiming to provide guidance toward the validation or qualification of AI tools within different scenarios and pathways Consolidate input from scientific experts, health authorities, and published guidelines

^a^ML: machine learning.

^b^PROM: patient-reported outcome.

^c^CDSS: clinical decision support system.

^d^CIG: computer-interpretable guideline.

^e^CAD: computer-aided design.

^f^AI: artificial intelligence.

## Discussion

This paper presents the key topics that were discussed as part of two international workshops on the current status of technological and data-driven innovations in cancer care. Key challenges and opportunities have been identified, and several recommendations have been made to facilitate the acceleration of progress in the data-driven management of cancer. The workshops presented the work that was being conducted in four Horizon 2020 EU–funded projects, namely BOUNCE, CATCH ITN, DESIREE, and MyPal. These projects provide a rich landscape of the challenges and opportunities of the current state of the art of new technologies in cancer care. The authors have presented these issues and discussed recommendations that can be used for further research as well as practical implementation of such tools in cancer.

## Abbreviations

ACC: accuracy
AI: artificial intelligence
AUC: area under the curve
CDSS: clinical decision support system
CIG: computer-interpretable guideline
CPG: clinical practice guideline
DSS: decision support system
EHR: electronic health record
ePRO: electronic patient-reported outcome
EU: European Union
FDA: Food and Drug Administration
H2020: Horizon 2020
ICT: information and communication technology
IEEE: Institute of Electrical and Electronics Engineers
IT: information technology
LSTM: long short-term memory
MD5: message-digest algorithm 5
mHealth: mobile health
ML: machine learning
NCCN: National Comprehensive Cancer Network
PROM: patient-reported outcome
SaMD: software as a medical device
